# Relationship Between Signals from Cerebral near Infrared Spectroscopy Sensor Technology and Objectively Measured Cerebral Blood Volume: A Systematic Scoping Review

**DOI:** 10.3390/s25030908

**Published:** 2025-02-03

**Authors:** Noah Silvaggio, Kevin Y. Stein, Amanjyot Singh Sainbhi, Nuray Vakitbilir, Tobias Bergmann, Abrar Islam, Rakibul Hasan, Mansoor Hayat, Frederick A. Zeiler

**Affiliations:** 1 Department of Human Anatomy and Cell Science, Rady Faculty of Health Sciences, University of Manitoba, Winnipeg, MB R3T 2N2, Canada; 2 Department of Biomedical Engineering, Price Faculty of Engineering, University of Manitoba, Winnipeg, MB R3T 2N2, Canada; steink34@myumanitoba.ca (K.Y.S.); amanjyot.s.sainbhi@gmail.com (A.S.S.); vakitbir@myumanitoba.ca (N.V.); bergmant@myumanitoba.ca (T.B.); islama9@myumanitoba.ca (A.I.); hasanr2@myumanitoba.ca (R.H.); frederick.zeiler@umanitoba.ca (F.A.Z.); 3 Section of Neurosurgery, Department of Surgery, Rady Faculty of Health Sciences, University of Manitoba, Winnipeg, MB R3T 2N2, Canada; mansoor.hayat@umanitoba.ca; 4 Department of Clinical Neuroscience, Karolinska Institutet, 17177 Stockholm, Sweden; 5 Pan Am Clinic Foundation, Winnipeg, MB R3M 3E4, Canada

**Keywords:** cerebral blood volume, cerebral near-infrared spectroscopy, NIRS, CBV

## Abstract

Cerebral blood volume (CBV) is an essential metric that indicates and evaluates various healthy and pathologic conditions. Most methods of CBV measurement are cumbersome and have a poor temporal resolution. Recently, it has been proposed that signals and derived metrics from cerebral near-infrared spectroscopy (NIRS), a non-invasive sensor, can be used to estimate CBV. However, this association remains vastly unexplored. As such, this scoping review aimed to examine the literature on the relationship between cerebral NIRS signals and CBV. A search of six databases was conducted conforming to the Preferred Reporting Items for Systematic Reviews and Meta-Analyses guidelines to assess the following search question: What are the associations between various NIRS cerebral signals and CBV? The database search yielded 3350 unique results. Seven of these articles were included in this review based on the inclusion and exclusion criteria. An additional study was identified and included while examining the articles’ reference sections. Overall, the literature for this systematic scoping review shows extreme variation in the association between cerebral NIRS signals and CBV, with few sources objectively documenting a true statistical association between the two. This review highlights the current critical knowledge gap and emphasizes the need for further research in the area.

## 1. Introduction

Cerebral blood volume (CBV) is defined as the volume of blood in a particular amount of brain tissue, usually measured in millimetres of blood per 100 g of brain tissue [1]. CBV can be used as an essential physiological indicator to evaluate the health and functionality of brain tissue [1]. In a healthy brain, it is observed that any small changes in CBV are accompanied by small changes in both the intracranial pressure (ICP), as well as the cerebral blood flow (CBF) [2]. During normal physiological conditions, the brain continuously regulates the diameter of blood vessels in an attempt to maintain constant CBF in the presence of dynamic systemic pressure [1]. Any failure to do so can affect CBV values, indicating potential dysregulation in the brain [1]. However, in a case where CBV may be abnormally high, such as an intracerebral hemorrhage, the increased blood volume in the brain can lead to pressure buildup and, therefore, an increased ICP [3]. On the other hand, when CBV is abnormally low, both decreases in ICP as well as the CBF can be seen [4].

There are many different methods of measuring CBV, including both continuous and intermittent methods or sensor sources. One method frequently used to measure CBV is magnetic resonance imaging (MRI). Measurement for MRI-based CBV sensory technology uses a contrast agent, which allows for measurements of CBV to occur with good spatiotemporal resolution [5]. Alternatively, blood-oxygen-level-dependent (BOLD) contrast methods can also be used during functional MRI (fMRI); however, this method has a poor temporal resolution [6]. The poor temporal resolution seen with fMRI is a result of limitations seen with the speed of the BOLD response [7]. About 6 s after neural stimulus, a peak is observed within the BOLD response, which is much slower than what is observed in neural processes, leading to a poor temporal resolution [7]. Another method used to measure CBV is through the use of radiolabelled indicators, where blood is withdrawn from the subject to mix indicators with the blood sample [8]. Then, the withdrawn blood is re-injected into the subject, and another sample of blood is taken in order to re-measure the radioactivity 5 min post-injection [8]. This way, the CBV can be calculated through the relation of radioactivity in the tissue and radioactivity of the 5-min reference blood sample [8]. Some other common methods and sensor sources of recording CBV include the use of single-photon emission computerized tomography (SPECT) imaging [9], dynamic susceptibility contrast (DSC) MRI [9], positron emission tomography (PET) [10], and determining changes in the total hemoglobin concentration (tHgB) through the measurement of oxygenated hemoglobin (OxHgB) and deoxygenated hemoglobin (deOxHgB) [11]. While many of these methods provide an accurate estimate of CBV, many of them are cumbersome in the equipment and personnel required, have a poor temporal resolution, and, in some cases, involve the use of radiation. Additionally, values for CBV obtained by these methods are often incomparable with values from another method of measurement [12].

The use of near-infrared spectroscopy (NIRS) sensory technology as a method of measuring cerebral physiology was first described in 1977 by Franz Jöbsis [13]. NIRS uses near-infrared (NIR) light of different wavelengths (usually 650–950 nm) as it is able to pass through the scalp and skull to reach the cerebral parenchyma and be absorbed by physiologic chromophores, such as OxHgB and deOxHgB [14]. By measuring the proportion of the emitted NIR light that is scattered, the modified Beer–Lambert Law can be used to estimate the concentrations of OxHgB and deOxHgB [14,15]. Other measures such as the tHgB and the regional cerebral oxygen saturation (rSO_2_) can also be calculated [14,15]. These measures are believed to be able to represent a surrogate measure for CBV, with a much higher temporal resolution than seen with previous measurement methods and sensors for CBV [14,16]. The penetrative characteristics of NIR light allows cerebral NIRS to be used as a non-invasive sensor to continuously monitor various aspects of cerebral physiology. On top of the non-invasive nature of NIRS, another benefit over traditional invasive continuous monitoring of cerebral physiologic processes is the ease of use, as the NIRS system can be utilized without the need for surgical skills. Moreover, cerebral NIRS systems cost a fraction of what single-use invasive devices cost, making them more cost-effective. 

There have been advancements in attempting to use NIRS to estimate CBF with some success in displaying a positive linear relationship between NIRS variables and changes in CBF [14]. There has been a steady increase in the number of papers discussing diffuse correlation spectroscopy (DCS), with a large number of these discussing the use of DCS in CBF estimation [17]. DCS produces real-time estimates of CBF through observing reflected NIR light in order to observe temporal fluctuations in blood flow [14]. Despite the interest in CBF estimations, it is just as important to look at the utilization regarding CBV as well. CBF differs from CBV in the fact that CBF is the volume of blood flowing through a particular amount of brain tissue in a period of time [18]. Both CBV and CBF are important physiological indicators to evaluate brain health [1,18]. There are some cases in which CBV would be clinically more useful to obtain. An example of this can be seen with strokes, as although CBF may better predict the outcome of brain tissue, CBV can be used to pinpoint salvageable brain tissue [19]. This is because in brain tissue where flow is obstructed, CBF values can be impacted to a higher degree than CBV values [19]. Both CBF and CBV have extreme clinical importance and should both be explored.

NIRS is a promising method for measuring cerebral physiologic processes not only because of its high temporal resolution but also due to its non-invasive and continuous nature. Despite the belief that NIRS signals can represent a surrogate for CBV, this association is unclear within the existing literature, with data scattered and difficult to compare. Therefore, the objective of this systematically conducted scoping review is to examine both human and animal studies to determine if there exists a relationship between cerebral NIRS signals and objectively measured CBV.

## 2. Methods

This systematic scoping review of the literature was conducted following the methodology described in the Cochrane Handbook for Systematic Reviews [20]. The information reported in this systematic scoping review conforms to the Preferred Reporting Items for Systematic Reviews and Meta-Analyses extension for Scoping Reviews (PRISMA-ScR) [20,21]. The completed PRISMA-ScR checklist is found in Appendix A. The objectives for this systematic scoping review’s search strategy were developed collaboratively by NS and FAZ, with help from KYS in the article filtering process.

### 2.1. Ethical Consideration

All articles examined and included in this systematic scoping review were from previously published journals and are assumed to have been screened by these journals. Therefore, specific ethics approval for this systematic scoping review was not required.

### 2.2. Search Question and Criteria for Inclusion and Exclusion

The question examined in this review is as follows: What are the associations between various NIRS cerebral signals and CBV? To be included in this review, articles needed to include an objective comparison between cerebral NIRS sensor signals and a CBV measure, with CBV data being either continuous or intermittent in human or animal studies. Of note, for objective comparison to cerebral NIRS signal sources, the CBV measures for included articles had to consist of a separate distinct method for true CBV determination (i.e., cerebral NIRS signals could not be internally compared to a CBV metric derived from the cerebral NIRS data itself). Articles that were excluded were non-English, not full-length, or did not present a direct statistical comparison between a cerebral NIRS-derived metric and CBV.

### 2.3. Search Strategy

Searches were conducted using BIOSIS, SCOPUS, EMBASE, MEDLINE, Global Health, and Cochrane Library, encompassing the period from the inception of each database until 13 June 2024. Search strings, which included terms and synonyms for brain, NIRS, and CBV, were constructed. Included in the appendix (Appendix B) is the detailed search strategy used for each database. Once a search was conducted using each database, the results were combined, and the papers were deduplicated to make an extensive list of potential articles.

### 2.4. Selecting Studies

After deduplication, the remaining articles were manually reviewed through a two-stage, two-reviewer approach. In the first stage, two reviewers (NS and KYS) independently screened the title and abstract of each article based on the inclusion and exclusion criteria. Articles that were included went to the second stage of screening, where both reviewers looked at the complete article and assessed it based on the inclusion and exclusion criteria. Any disagreements between the reviewers were resolved by a third party (FAZ). The reference sections of the included articles were then screened to ensure the inclusion of all relevant articles in this systematic scoping review.

### 2.5. Data Collection

Characteristics were recorded from each article included in this systematic scoping review, encompassing the patient/subject information, general study information, and the results. Patient/subject information included what patients/subjects were used, as well as their sample size for the study. General study information included how CBV was recorded, what cerebral NIRS device was used, the NIRS signal that was examined, and the experimental conditions that were set during the study. Other information regarding the association between the cerebral NIRS signal and the measurement of CBV, as well as the limitations of the different studies, were also obtained.

### 2.6. Statistical Analysis

Considering the highly heterogeneous nature of this study as well as the literature in cerebral physiology investigated, no formal meta-analysis was performed.

### 2.7. Bias Assessment

Given that the primary aim of this review was to conduct a comprehensive scoping review of the available literature, a formal bias assessment was not warranted.

## 3. Results

### 3.1. Search Strategy and Results

**Figure 1:**
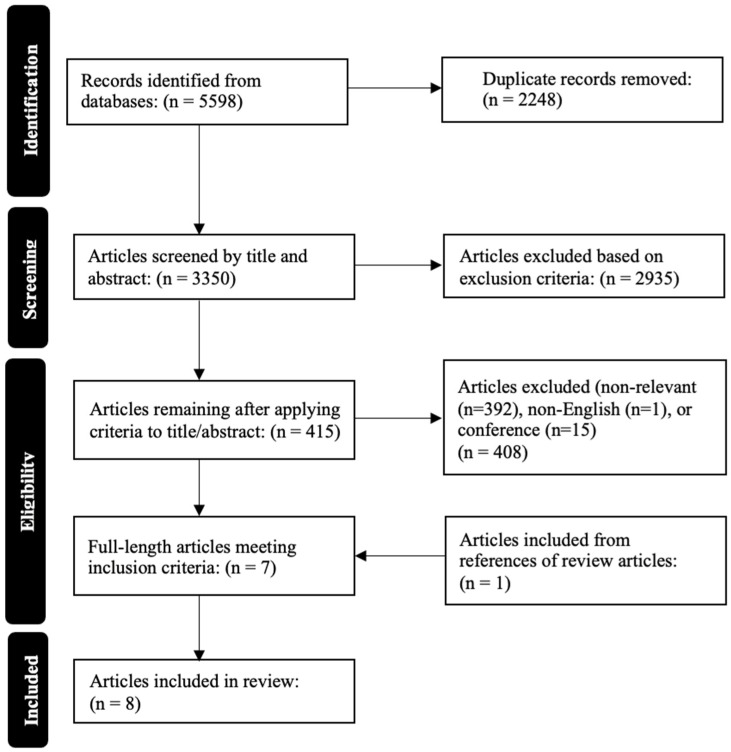
PRISMA flow diagram of the systematically conducted scoping review.

The overall results for the search and filtration processes are shown in Figure 1, using a PRISMA flow diagram. Overall, six databases were searched (BIOSIS, SCOPUS, EMBASE, MEDLINE, Global Health, and Cochrane Library), as well as the reference sections of the articles that met the initial inclusion criteria. A total of 5598 articles were identified through the initial search, with 2248 of these articles being excluded through the deduplication process. This led to 3350 articles being screened by the title and abstract based on the inclusion and exclusion criteria. Of these articles, 2935 articles were excluded for not meeting the set inclusion criteria, leaving 415 articles that were screened by examining their full text. Of these articles, 408 of them were excluded for not meeting the inclusion criteria, which left 7 articles from the initial search that were included in this review. Through the examination of the reference sections of these included articles, an additional article was included, making a total of eight articles included in this review.

### 3.2. Study Demographics

Of the eight articles included in the final review, four were human-based studies, and four were animal-based. From the studies that were animal-based, three studies were performed on newborn piglets [22,23,24], while the other one was performed on fetal lambs [8]. All of the studies that were human-based were performed on adults (age 18 and older) [25,26,27,28]. One of these studies that used adult humans incorporated patients who required contrast-enhanced MRI for clinical reasons and were able to consciously decide to partake in the study [25]. The other three articles incorporated the use of consenting healthy adult human volunteers with an age range from 22 to 51 years old [26,27,28]. Individual sample sizes range from 3 to 18 [8,22,23,24,25,26,27,28]. Characteristics for the individual studies are summarized in Table 1.

### 3.3. NIRS Conditions and Variables

Out of the eight studies included in this systematic scoping review, four used commercially available NIRS devices to obtain their measurements: the Hamamatsu NIRO-500 [8,27], the Critikon Cerebral RedOx Monitor-2020 [25], and the Hamamatsu TRS-10 system [28]. The other four studies utilized prototype or research devices [22,23,24,26]. The most common cerebral NIRS signals investigated in these studies were concentrations of OxHgB and deOxHgB [8,22,23,25,27,28]. The tHgB concentration can be calculated using the following equation [29]:

tHgB = OxHgB + deOxHgB

As described in some of the articles [8,22,23,25,27,28], the tHgB can provide an estimate for the CBV through the following equation:

CBV = (Δ [OxHgB] − Δ [deOxHgB])/(2 × ΔSaO_2_ × tHgB × *R*),

where ΔSaO_2_ is the change in oxygen saturation, and *R* is the cerebral to large vessel hematocrit ratio [30]. In this equation, the numerator results in a value for the change in the hemoglobin concentration, which is directly proportional to the amount of blood in the area [30]. *R* is used to correct for the difference in the hematocrit between cerebral vessels and large vessels [30]. Using these values, as well as the tHgB, the true value for CBV can be estimated.

Out of the studies included in this systematic scoping review, two studies did not include either OxHgB or deOxHgB; instead, they used NIRS alongside indocyanine green (ICG) dye dilution to measure regional cerebral blood volume (rCBV) through the combination [24,26]. In a study by Keller and colleagues, this was achieved by using the following equation:

$${r{CBV}_{NIRS}=c}_{ICG}^{tissue}/c_{ICG}^{blood},$$

where $c_{ICG}^{tissue}$ is the concentration of ICG in the illuminated volume of tissue, and $c_{ICG}^{blood}$ is the concentration of ICG in cerebral blood, both measured using NIRS [26].

Despite many of the same variables being measured in the articles included in this systematic scoping review, the context in which data for these variables were obtained differed between the articles. In the study conducted by Barfield and colleagues, this involved altering the fraction of inspired oxygen before recording data [8]. In another study conducted by Brun and colleagues, the levels of both oxygen and carbon dioxide in the blood were altered, as hypocapnia, hypercapnia, hypoxemia, and hypoxemia with hypocapnia were investigated [22]. In another two studies, measurements were made in three different conditions including rest, hypercapnia, and hypocapnia [24,27]. Similarly, in a study completed by Klassens and colleagues, three conditions were also explored; however, they included a rest state, a state of mild hypercapnia, and a state of severe hypercapnia [23]. Keller and colleagues used variables measured during varying levels of positive end-expiratory pressure through the use of continuous positive airway pressure (CPAP) breathing [26]. Lastly, in a study performed by Ohmae and colleagues, measurements were taken at rest, and after the administration of 1000 mg of acetazolamide [28].

### 3.4. CBV Techniques and Measurement

Over the eight articles included in this systematic scoping review, there is an extensive range of techniques used to measure CBV. In two of the articles included, CBV was measured through the use of radiolabelled indicators [8,22]. In a study by Brun and colleagues, erythrocytes labelled with ^99m^Tc-pertechnetate were injected into a piglet, where a scintillation detector was used to continuously record the activity over the head [22]. In another study, fetal blood was withdrawn and labelled with both chromium 51 and RISA (^125^I-labelled albumin) [8]. The radioactivity of ^51^Cr-RBC and ^125^I-RISA in the mixture was counted before it was injected back into the fetal lamb [8]. Further blood samples were then withdrawn at 1, 2, 3, 4, and 5 min post-injection in order to be re-measured [8]. Two other studies used MRI as a method of determining CBV, both requiring a bolus injection of a paramagnetic contrast agent for contrast-enhanced MRI [25,26]. Other methods of determining CBV included the use of PET, computed tomography (CT), or ultrasound which required the use of a contrast agent (CA) [23,24,27,28].

As seen with the NIRS measurements, the states in which data for CBV was collected differed between articles discussed in this systematic scoping review. For the majority of the articles, cerebral NIRS signals and CBV were collected in the same state. However, in one of the articles, while measurements for NIRS occurred during a state of lessened oxygen consumption, the collection of CBV data occurred during baseline [8].

### 3.5. NIRS Association and Agreement with CBV

Overall, the available body of literature used for this systematic scoping review shows variation in the association between cerebral NIRS signals and CBV. Many of the studies calculated the statistical significance between CBV values obtained by the different measurement methods, with a many papers indicating correlations between NIRS variables and CBV [22,23,24,25,26,27,28], while others failed to show any correlation at all [8]. In one study, it was shown that values for CBV obtained with NIRS were statistically different and did not correlate with values for CBV obtained using radiolabelled indicators [22]. When comparing NIRS-based CBV values to MRI-based CBV values, Keller and colleagues found that the two methods positively correlated with one another, despite there being significant differences in values obtained as 83% of the values increased or decreased simultaneously between the two methods of measuring CBV [26]. In another study, it was shown that CBV values obtained using both NIRS and radiolabelled indicators were not statistically different from one another (*p* > 0.05) [8]. Similarly, Wolf and colleagues also found that values obtained for CBV across two methods were not statistically different from one another; however, in this study, MRI was used to measure CBV instead of radiolabelled indicators [25]. Despite CBV values between the two methods being very similar, it was determined that the methods were not correlated [*r* = −0.297 (Pearson) and *r* = −0.266 (Spearman)] as both tests of correlation failed to reach statistical significance [25]. In a study performed by Rostrup and colleagues, it was determined that there was a moderate positive correlation between CBV values obtained through NIRS and PET (*r* = 0.56, *p* = 0.05), despite CBV values obtained through NIRS being a much smaller magnitude [27]. In another study performed by Ohmae and colleagues, it was also determined that values for CBV obtained through both NIRS and PET were positively correlated with one another; however, there was a large range in the strength of correlation (from *r* = 0.050 to *r* = 0.859) as it depended on both the volume of interest and the optode distance [28]. Similarly, Klaessens and colleagues also determined there to be variation in the correlations observed between CBV values, except, here, CBV was measured by NIRS and ultrasound contrast agent densitometry [23]. Here, the strength of correlation (ranging from *r*^2^ = 0.02 to *r*^2^ = 0.83) was influenced by both the level at which CBV was measured, as well as which hemisphere was being measured [23]. Lastly, in an experiment conducted by Brown and colleagues, it was determined that values for CBV obtained through NIRS and CT were not significantly different from each other [*p* > 0.1 (two-way ANOVA test)], with the CBV values obtained from the two different methods being positively correlated with one another (*r*^2^ = 0.75) [24].

## 4. Discussion

In this systematic scoping review, we sought to determine the relationship between cerebral NIRS sensor signals and objectively measured CBV. Across the eight different studies included in this review, it proved challenging to determine this relationship, as results presented within the included articles contradicted one another.

Through this systematic scoping review, it became clear that very few studies exist that explore objective comparisons between cerebral NIRS sensor signals and CBV, as only eight studies were included in this review. This is alarming, as the idea that values obtained through NIRS sensor technology can be used to represent values of CBV has become generally adopted in the community. This idea has been commonly used in the literature surrounding cerebral physiology and non-invasive cerebral measurement, despite the lack of evidence that supports this relationship. This is further emphasized by the number of subjects included in the studies exploring the relationship between cerebral NIRS sensor signals and CBV, as the largest sample size included in this review was 18 [8]. With the fact that there exists a small number of studies and that these studies include small sample sizes, further objective comparisons between NIRS and CBV need to be made before the notion that values obtained through NIRS can represent CBV is adopted.

Two separate studies included in this systematic review determined a significant difference in values obtained for CBV using NIRS sensor technology and values for CBV obtained using a gold-standard method of measurement [22,26]. One reason why these differences were observed could be attributed to the idea that values obtained through NIRS are less affected by volume changes in the veins than when values were obtained through radioactively labelled erythrocytes [22]. This is because the optical field of the NIRS device included a large portion of the brain, without including either the superior sagittal sinus or the venous plexus [22]. In contrast, CBV values obtained through the use of radioactively labelled erythrocytes could have been impacted at the venous plexus, as a result of the lead shielding [22]. Another reason why significant differences between CBV values were observed could be due to the setting in which CBV values measured using cerebral NIRS sensors were obtained, as values obtained using the NIRS instrument could be impacted by both the emotional and physiological states of the volunteer, including anxiety and blood pressure differences [26]. Despite there being significant differences in CBV values, Keller and colleagues determined there to be a positive correlation between CBV values obtained from the different methods [26].

In another two studies included in this review, it was determined that the CBV values obtained using NIRS were not statistically different from the CBV values obtained using a separate method of measurement [8,25]. However, in the paper by Wolf and colleagues, the correlation between CBV measurements failed to reach any sort of statistical significance [25].

In the other four studies included in this review, it was determined that a positive correlation exists between CBV values obtained through NIRS and CBV values obtained through a secondary mode of measurement, whether this be PET, CT, or ultrasound CA [23,24,27,28]. However, two of these studies noted large ranges in the correlations observed as a result to differences in the optode distance as well as differences in the area being measured [23,28]. Despite the promising findings of a positive correlation between CBV measured through NIRS and a secondary mode of measurement, the small sample sizes included in these studies limit the generalizability of these findings, as the largest sample size among this group is 6 individuals [23,24,27,28].

Due to the variation in results obtained from the eight articles, determining a relationship between cerebral NIRS signals and CBV was challenging. As mentioned, some papers found statistical differences between values for CBV while using two different methods, while others did not. Despite this, CBV values obtained using NIRS sensor technology can potentially provide an estimate of the true value of CBV. Regardless, additional research should be conducted to determine the true relationship between cerebral NIRS sensor signals and CBV.

The monitoring of cerebral physiologic processes in large mammals and humans primarily uses invasive sensor technology, leading to significant limitations [31]. These limitations can include potential complications during the procedure [32], the expertise required to install devices to monitor the devices [33], and also the cost associated with these invasive devices [34]. NIRS provides a potential solution to these limitations as variables including OxHgB and deOxHgB can be obtained non-invasively [14,15]. These variables not only have the potential to represent measurements of CBV but also other cerebral physiologic variables such as ICP [14,16]. Despite these advancements, this systematic scoping review confirms that these techniques remain experimental, and additional research into the specific relationships is needed.

### 4.1. Limitations of the Literature

The literature examined during this systematic scoping review has several limitations. Articles included in this review display vast heterogeneity in the experimental designs, subjects used, and methods of measurement, making the true relationship between cerebral NIRS signals and CBV challenging to establish.

One limitation observed in the available literature was the inclusion of small sample sizes. In many cases, the included sample size was very small, with one study having a sample size of three individuals [23], one study including five individuals [27], and three studies including a sample size of six individuals [22,24,28]. A small sample size cannot only affect the reliability of the results but also impact generalizability.

Leading to the second limitation of using different subjects to obtain measurements from two different methods to be compared with each other, in which both the accuracy and generalizability of the results could be limited. In a study completed by Barfield and colleagues, different subjects were used between NIRS measurements and measurements of CBV [8]. This limits the accuracy of comparison between the obtained values, as differences in CBV values between subjects can incorrectly be placed on inaccuracies between the two measurements of CBV.

Another limitation observed in some of the included studies is that some of the measurements for CBV and NIRS did not occur simultaneously [8,25,26]. Similarly to what can be seen when comparing values obtained from different subjects, differences in CBV values across time can be mistaken for inaccuracies in measurement methods.

Another limitation that could be observed in some of the included studies is the idea of cerebral sensitivity. Cerebral sensitivity can vary between individuals, leading to potential differences in sensitivity to environmental stimuli [35]. This could lead to differences in values obtained between subjects, despite the experimental design remaining the same. In a few of the studies included in this review, subjects went through severe trauma before the measurement of CBV, such as placing the NIRS optodes directly on the skull or making an artificial fontanel [8,22,23]. The idea of cerebral sensitivity could be used here, as responses to this trauma could significantly differ between individuals.

Alternatively, studies where NIRS optodes were placed directly onto the skull may be more reliable with respect to the idea of extra cerebral contamination [8,22]. One of the potential drawbacks of NIRS sensor technology is that extra cerebral contamination could impact the values recorded [36]. Different hemodynamics of the scalp such as oxygenation and blood flow could interfere with cerebral signals [36]. In the studies that utilized NIRS on top of the scalp, extra cerebral contamination is a possibility and could have potentially impacted their values [24,25,26,27,28].

Lastly, articles identified by this review generally failed to perform comparative analysis and simply investigated whether or not a statistical difference was observed between values obtained using NIRS and a secondary mode of measurement. While some papers did provide simple correlation analysis, they failed to assess a trend in the bias [23,26,27,28].

### 4.2. Limitations of the Review

Additionally, there were limitations exclusive to this review. The initial inclusion and exclusion criteria of this systematic review could exclude some studies with information that could help find the relationship between cerebral NIRS signals and CBV. For example, this review only investigated full-length English studies that were published prior to 13 June 2024. Any study that did not fall within these guidelines was excluded from this review and not included in our analysis of the relationship between cerebral NIRS signals and CBV.

Among the exclusion criteria was the removal of any study that used NIRS as an internal comparison for CBV. This specifically included studies where the initial value for CBV was derived through NIRS, and then subsequent CBV values were compared to the initial measurement. This was conducted to prevent internal confirmation bias, allowing the review to focus on the direct comparison between cerebral NIRS variables and an external objective CBV measure. Despite this, there is the possibility that some studies with potentially relevant comparisons and relationships were excluded in the process.

### 4.3. Limitations of NIRS Sensor Technology

Despite the promising idea of measuring different cerebral physiologic processes, such as CBV, using NIRS sensor technology, there are a few limitations to the technology itself that could potentially inhibit this idea. First is the idea of extracranial contamination, in which it is often difficult to differentiate between signals from cerebral tissue and signals from extracerebral tissue, especially when the sensor is placed on top of the scalp [37]. This limitation can be a result of the NIRS sensor only utilizing one long distance channel, without a short reference channel, and therefore collecting a mixture of extracranial and cerebral signals. This is further complicated by the fact that many commercially available NIRS platforms with both short- and long-distance emitter–detector spacing keep such extracranial signal removal methods protected behind black-box proprietary algorithms.

Second, a potential limitation of NIRS sensor technology is the fact that skin melanin can have an impact on data obtained through NIRS [38]. This is a result of melanin in the skin absorbing the NIR light before it has the chance to penetrate deeper and be absorbed by physiological chromophores [38]. This can further impact the values obtained through the modified Beer–Lambert Law, as the amount of light scattered could be different between individuals as a result of differing concentrations of melanin in the skin [14,15,38].

Thirdly, despite NIRS sensor technology being considered to have a higher temporal resolution than other methods of cerebral physiologic measurement, many systems still possess critical limitations [14,16,39]. When compared with EEG, the temporal resolution of NIRS is much lower as EEG typically utilizes sampling rates at a much higher magnitude than what is observed in NIRS sensory technology [39,40]. This becomes critical particularly in the absence of higher frequency NIRS data that contain cardiac pulse waveform data, a common issue with commercially available systems in human literature. The lack of cardiac cycle waveform characteristics can limit the ability to accurately model CBV using NIRS data streams.

Fourth, the spatial resolution of NIRS sensor technology could be another potential limitation. Unfortunately, NIR light is unable to penetrate to deeper brain structures when it is utilized at a standard SDS, limiting the spatial resolution of the technology [40]. Similarly, many commercial NIRS devices lack multi-channel capacity, impacting cerebral cortical topographic spatial resolution and limiting potential CBV assessments to single small regions. Both of these aspects of spatial resolution provide a challenge when comparing NIRS data streams to more standard CBV measures obtained from multiple simultaneous regions of interest (both superficial/deep and multiple cortical regions), as seen with advanced neuroimaging techniques.

Lastly, the topic of artefacts could be a potential limitation of NIRS sensor technology. Any subject movement, sensor adherence issues or data acquisition/connection errors and introduce significant artefact segments into otherwise clean data streams [41]. Such artefacts need to be adequately managed prior to analysis of data, through either manual or semi-automated/automated means. Furthermore, proper artefact identification and removal lead to missing data segments within the NIRS data streams, which need to be addressed. Interpolation techniques of such missing segments remain a major knowledge gap in cerebral physiologic signal analysis [41].

### 4.4. Future Recommendations

Future efforts should be conducted to better define the relationship between continuous NIRS signals and CBV. The available body of literature included in this systematic scoping review provides hope that cerebral NIRS signals, such as OxHgB and deOxHgB, can provide an estimate for CBV values. With further study into the correlational relationships of NIRS signals and CBV, the true relationship between the two can potentially be determined. This is important as the idea that CBV can be represented through NIR values has become commonly used in the literature surrounding cerebral physiology and non-invasive cerebral measurement. Without sound evidence, further research into this topic is needed.

One recommendation for future study into the relationship between continuous NIRS signals and CBV would be to utilize a continuous measure of CBV. Within the studies included in this review, there were various different measures of CBV. Since NIRS signals look at continuous data, it is best to use a continuous source of CBV measurement, so that comparisons can be made in real time. Regarding limitations to the NIRS sensor technologies, advancements and alterations to the applied technology itself could lead to a more accurate representation of cerebral physiology. Firstly, adherence to the utilization of both short- and long-channel emitter–detector separation at each region of interest is key to facilitate capturing true cerebral signal sources. This should be ideally conducted using systems which record both short- and long-distance data at each region (including absorption spectrum and/or optical density data) so that scrubbing extra-cranial contamination can occur in a transparent and reproducible manner. Similarly, ensuring the use of NIRS platforms which only register true signal sources in the presence of a “delta” or change in absorbance over time, will ensure static non-physiologic NIRS absorbances related to melanin are eliminated as a potential confounder in recorded signal sources. Next, to address temporal resolution challenges, future studies need to utilize NIRS systems which capture full waveform data with cardiac cycle features, which would mean ideally a minimum of 50 Hz sampling frequency to facilitate time-frequency domain analytic methods of CBV modelling. Cortical spatial resolution challenges can easily be mitigated through used modern multi-channel systems that facilitate short/long emitter–detector recording at several regions of interest simultaneously. Several research grade systems, currently available on the market, address all of these above NIRS limitations and provide the ability for external validation of future studies by avoiding “one-off” lab-built NIRS solutions that are challenging to recreate. Regarding artefacts, the development of real-time artefact management would be beneficial, allowing for a more accurate measurement of the present cerebral physiology. Work here will require a ground-up build of a reliable layered sequential approach to the various NIRS artefacts encountered in practice. Such pipelines are under development by our group.

Another recommendation for future study would be to use different modalities of NIRS to investigate the relationship between NIRS signals and CBV. Within the studies investigated in this review, two specified that continuous wave (CW) NIRS was utilized [23,27], while one review specified using time domain (TD) NIRS [28], with the other studies not specifying the exact modality used [8,22,24,25,26]. There are many different modalities of NIRS sensor technology that could potentially reduce some of the potential limitations. One of the most commonly used modalities of NIRS sensor technology is CW-NIRS, as it has widespread commercial availability [14]. CW-NIRS utilizes only one wavelength at a time and utilizes the amount of light absorbed by the tissue to determine concentrations of a particular physiological chromophore [14]. This poses potential limitations in both the spatial and temporal resolution as the degree of light scatter is assumed to be constant for the particular wavelength of NIR being used, despite the potential tissue differences that exist [42]. The use of TD-NIRS increases the temporal resolution, which is a potential limitation of CW-NIRS technology. TD-NIRS utilizes very short pulses of NIR light in order to measure how long it takes for the light to pass through a particular tissue [14]. Another modality that could be utilized to increase the temporal resolution could be DCS. DCS is able to produce real-time estimates of CBF through observing reflected NIR light in order to observe temporal fluctuations in blood flow [14] and has recently been extensively studied for its ability to estimate CBF, and it would be interesting to see if estimations for CBV can be made as well [17]. On the other hand, frequency domain (FD) NIRS can increase the spatial resolution that is a potential limitation of CW-NIRS technology. Since FD-NIRS technology modulates the intensity of the NIR light, both the changes in light intensity as well as the phase shift in the light allow for a better spatial resolution [14]. The utilization of TD-NIRS and FD-NIRS would not only increase the spatial and temporal resolutions, respectively, but also the depth selectivity. The utilization of either short pulses of NIR by TD-NIRS devices, or the altering the intensity of NIR by FD-NIRS devices provide more information regarding how the NIR interacts with different tissue compositions [43].

This knowledge is also important as it can pave the way for the potential use of NIRS as a non-invasive tool to measure cerebral physiologic processes. ICP is a surrogate for CBV, with the caveat that measurement of ICP often requires invasive measurement, including parenchymal or cerebrospinal fluid-based devices [14,16,31]. Through further exploring the relationship between cerebral NIRS signals and CBV, future studies can then use this information to extend these relationships and methods to investigate the use of NIRS to measure other cerebral physiologic processes, such as ICP.

## 5. Conclusions

A search across six databases was conducted for this systematic scoping review, which investigated the relationship between cerebral NIRS signals and CBV. This search resulted in very few existing literature sources documenting an objective comparison between cerebral NIRS signal sources and CBV. The results varied between the few identified studies, highlighting the current critical knowledge gap in our understanding of the relationship between cerebral NIRS and CBV. Future work is necessary in this area of cerebral physiology to delineate this critical relationship because it acts as a foundational step in determining the relationship between cerebral NIRS signals and other cerebral physiologic processes. Also, it will provide the needed confidence in the future use of NIRS as a non-invasive tool to measure these cerebral physiologic processes.

## Figures and Tables

**Table 1:** Summary of articles examining relationship between cerebral NIRS signals and CBV.

Article	Study Subjects	Number of Subjects	Measure of CBV	NIRS Device, Wavelength, and SDS	NIRS Signal Examined	Experimental Conditions	Association Determined	Study Limitations
Brun et al. (1997) [22]	Healthy newborn piglets	11 (6) *	CBV measured by using radiolabelled erythrocytes	Radiometer Prototype Instrument at four different wavelengths (774, 806, 845, and 910 nm). The optodes were applied directly to the skull symmetrically on each side.	CBV_NIRS_ (A function of total Hb)	During the experiment, data were collected every 0.5 s Measurements were taken during periods of hypocapnia, hypercapnia, hypoxemia, and hypoxemia with hypocapnia Hypoxemia was induced by lowering the amount of oxygen in the inspired air Both hypocapnia and hypercapnia were induced by changing the ventilation frequency and the minute volume	The average value for CBV measured in the normal state when using NIRS was 0.49 ± 0.04 mL/100 g. This value was significantly less than the value of CBV when using radioactively labelled erythrocytes (0.96 ± 0.04 mL/100 g) During the experiment, changes in CBV from state to state were uneven. When measuring CBV by radioactively labelled erythrocytes there was a significant difference (*p* = 0.03) in CBV values among states; however, this was not observed during a pairwise analysis of the states. Similar trends were seen when CBV was measured by NIRS, except during the hypocapnic states. CBV measured by NIRS was highest during the hypoventilation state (0.45 ± 0.09 mL/100 g), where as CBV measured by radioactively labelled erythrocytes was highest during the hypoxemia and hypoventilation state (1.08 ± 0.2 mL/100 g) Changes seen in CBV values were larger when measured by NIRS Values obtained for CBV using NIRS and values obtained for CBV while using radiolabelled indicators did not correlate with each other	Small sample size During hypoxemia and hypocapnia there could have been large volume changes occurring in the veins. This would potentially have a larger impact on values obtained when using radioactive measurements, than when using NIRS.
Barfield et al. (1999) [8]	Healthy fetal lambs	18	CBV measured by using radiolabelled red blood cells and radiodinated serum albumin	Hamamatsu NIRO-500. The optodes were applied directly to the skull symmetrically on each side over the temporal region. Wavelength is not specified.	CBV_NIRS_ (Using estimates of oxygenated Hb and deoxygenated Hb)	Group of healthy fetal lambs of Merino-Border Leicester cross Measurements for CBV_NIRS_ were taken at lowered SaO_2_ values (an 8–12% reduction in inspired oxygen) Measurements of CBV using radiolabelled indicators were conducted at baseline	Values for CBV measured using NIRS and the radiolabelled indicators were not statistically different (*p* > 0.05) when using a Mann–Whitney rank sum test. Values for CBV when using radio labels averaged 2.5 ± 0.2 mL/100 g for both the double indicator (^51^Cr-RBC and ^125^I-RISA) and the ^125^I-RISA single indicator, while the value for CBV when using the ^51^Cr-RBC single indicator was 2.5 ± 0.4 mL/100 g Values for CBV when using NIRS averaged 2.5 ± 0.2 mL/100 g	Measurements of the two methods were not taken simultaneously Measurements of the two methods were not performed in the same subjects Measurements of the two methods were performed in different conditions No bias assessment performed
Wolf et al. (1999) [25]	Healthy adult human patients	10	CBV measured by contrast enhanced MRI	Critikon Cerebral RedOx Monitor-2020 using a sensor with two receiving channels for different distances. Wavelength is not specified.	CBV_NIRS_ (A function of total Hb)	Group of healthy adult humans (6 F/4 M) Measurements of CBV_NIRS_ were taken before and after patients were in the MRI machine Measurements for CBV_MRI_ were taken at baseline over a period of 2 min	Mean values obtained for CBV_NIRS_ and CBV_MRI_ were not statistically different when a paired sample *t*-test and Wilcoxon signed rank test were performed. Mean CBV_NIRS_ was 8.6 ± 1.3 mL/100 g and the mean CBV_MRI_ was 7.1 ± 2.5 mL/100 g The average difference across measurements for CBV_NIRS_ was 11.5% Methods for obtaining values of CBV_NIRS_ and CBV_MRI_ were not correlated, both Pearson and Spearman correlation testing failed to reach statistical significance	Small sample size Measurements of the two methods were not taken simultaneously No bias assessment performed
Rostrup et al. (2002) [27]	Healthy adult volunteers	5	CBV was measured by PET	NIRO 500, Hamamatsu (CW NIRS) at four different wavelengths (775, 826, 850, and 910 nm). Optodes were placed on the left side of the forehead, with distances ranging from 3.5 to 4.7 cm	CBV_NIRS_ (A function of total Hb from oxygenated Hb and deoxygenated Hb)	Group of healthy adult humans (1 F, 4 M) Volunteers’ heads were stabilized to prevent movement during scans. Measurements using PET were conducted in two blocks, one consisting of three CBF scans, and the other consisting of three CBV scans. Each scan was carried out at a different condition (rest, hyperventilation, and hypercapnia). For CBV measurements, the subject inhaled C^15^O gas at each condition Measurements using NIRS were performed at three different conditions (rest, hyperventilation, and hypercapnia). NIRS recordings were continuous throughout the experiment	During the hypercapnia stage, there was an average increase of 29% in CBV values (*p* < 0.05). No statistical difference was seen between CBV values during rest and hypocapnia Average values for CBV_NIRS_ were 0.071 ± 0.073 mL/100 g at rest, 0.230 ± 0.074 mL/100 g during hypercapnia, and −0.074 ± 0.100 mL/100 g during the hyperventilation stage Average values for CBV obtained through PET were 5.5 ± 0.74 mL/100 g at rest, 7.0 ± 0.96 mL/100 g during hypercapnia, and 5.7 ± 1.00 mL/100 g during the hyperventilation stage Values for CBV obtained through NIRS and PET were similar to each other, except that NIRS values were of a much smaller magnitude There was a moderate positive correlation between CBV values obtained through NIRS and PET (*r* = 0.56, *p* < 0.05)	Small sample size No bias assessment was performed Some of the images went through a visual comparison, as it was not possible to coregister all of the images using the same template
Brown et al. (2002) [24]	Newborn piglets	6	CBV was measured by CT	Research Device at wavelengths ranging from 600 to 980 nm. Two optodes were placed on the head of the piglet, 3 cm apart from each other.	CBV_NIRS_ (A function of the absolute concentration of ICG in the tissue)	Group of newborn piglets were anesthetized prior to the experiment Measurements using CT were conducted at three conditions: hypocapnia, normocapnia, and hypercapnia. Using a CT perfusion software package, a functional map of CBV from four different slices was made. Measurements using NIRS were done at three conditions: hypocapnia, normocapnia, and hypercapnia	Using a two-way ANOVA test, there was no significant difference between CBV values obtained through CT and NIRS (*p* > 0.1) CBV values obtained using NIRS and CT were positively correlated with each other (*r*^2^ = 0.75)	Small sample size For 2 of the 6 piglets, NIRS and CT values were only obtained at two of the three conditions
Keller et al. (2003) [26]	Healthy adult volunteers	11	CBV measured by contrast enhanced MRI	Research device at three different wavelengths (905, 850, and 770 nm). Four diodes were used, with the emitter and the detector being placed 5 cm apart.	CBV_NIRS_ (As a ratio of ICG in illuminated volume of tissue to the concentration of ICG in cerebral blood)	Group of healthy adult humans (6 F, 5 M) Measurements were taken at varying levels of positive end expiratory pressure using a CPAP machine Measurements using NIRS were performed during day 1 of the experiment, whereas measurements using MRI were performed on days 2 and 3	Values for CBV_NIRS_ were 2.6 ± 1.0 mL/100 g while values for CBV_MRI_ were 29.4 ± 7.4 mL/100 g All of the differences determined between CBV_NIRS_ and CBV_MRI_ were within 2 standard deviations (± 15.6 mL/100 g) 83% of values for CBV_NIRS_ were positively correlated to the corresponding values for CBV_MRI_	Measurements of the two methods were not taken simultaneously
Klaessens et al. (2005) [23]	Newborn piglets	3	CBV measured by ultrasound contrast agent densitometry (Sonovue). AUTC was considered to be a relative measure of total CBV, where GLp is an alternative measure of CBVI	Research Device (CW NIRS) at three different wavelengths (767, 850, 905 nm). Optodes were separated at distances ranging from 3.5 to 6 cm.	CBV_NIRS_ (A function of total Hb from oxygenated Hb and deoxygenated Hb)	Piglets (3 M) were anesthetized, paralyzed and ventilated before the experiment began Measurements using ultrasound CA were performed during three conditions: normocapnia, mild hypercapnia, and severe hypercapnia. These conditions were held at 10 min each in order to reach a stable condition, where the CA would be injected. Measurements using NIRS were conducted for both hemispheres at three conditions: normocapnia, mild hypercapnia, and severe hypercapnia	For the right hemisphere, correlations (*R*^2^) between GL_p_ and CBV_NIRS_ were as follows: 0.83 (*p* < 0.05) at the surface level, 0.74 (*p* < 0.05) at the inner level, and 0.24 (*p* > 0.05) at the vessel level. Correlations between AUTC and CBV_NIRS_ were as follows: 0.64 (*p* < 0.05) at the surface level, 0.54 (*p* < 0.05) at the inner level, and 0.02 (*p* > 0.05) at the vessel level For the left hemisphere, correlations (*R*^2^) between GL_p_ and CBV_NIRS_ were as follows: 0.72 (*p* < 0.05) at the surface level, 0.67 (*p* < 0.05) at the inner level, and 0.16 (*p* > 0.05) at the vessel level. Correlations between AUTC and CBV_NIRS_ were as follows: 0.54 (*p* < 0.05) at the surface level, 0.46 (*p* < 0.05) at the inner level, and 0.04 (*p* > 0.05) at the vessel level	Small sample size No bias assessment performed Before the experiment, an artificial fontanel was made in an attempt to mimic a newborn human
Ohmae et al. (2006) [28]	Healthy adult volunteers	6	CBV was measured by PET	TRS-10 system, Hamamatsu (TD NIRS) at three different wavelengths (761, 791, and 836 nm). SDS is not mentioned.	CBV_NIRS_ (A function of total Hb)	Group of healthy adult humans (6 M) Measurements using PET were performed both at rest and in a loading state, which occurred 20 min after administration of 1000 mg of acetazolamide There were three VOIs for PET images. VOI_1_: included extracerebral tissues (scalp and skull). VOI_2_: included grey matter region. VOI_3_ included both grey and white matter regions Measurements using TRS were performed while volunteers were in either a rest state or a loading state. Optode spacings were changed in sequence between four light irradiation points, with acquisition times of 10 s, 20 s, 30 s, and 120 s. TRS measurements were performed during the entirety of the PET measurements.	During the rest state, TRS values for CBV at different optode spacings were 2.7 ± 0.4 cm^3^/100 g (2 cm), 3.0 ± 0.2 cm^3^/100 g (3 cm), 3.0 ± 0.3 cm^3^/100 g (4 cm), and 2.7 ± 0.3 cm^3^/100 g (5 cm). These values increased about 6% during the loading state. During the rest state, PET values for CBV among the different VOIs were 2.6 ± 0.4 cm^3^/100 g (VOI_1_), 4.4 ± 0.9 cm^3^/100 g (VOI_2_), and 4.0 ± 0.7 cm^3^/100 g (VOI_3_). During the loading state, values for CBV in VOI_2_ and VOI_3_ increased by about 10%; however, no significant differences were seen in VOI_1_ Values obtained for CBV through TRS and PET were positively correlated with each other; however, the strength of the relationship depended on both the optode distance and VOI used. At 2 cm spacing, *r*^2^ was 0.601 (*p* < 0.01) for VOI_1_ and 0.535 (*p* < 0.01) for VOI_2_. At 3 cm spacing, *r*^2^ was 0.410 (*p* < 0.05) for VOI_1_ and 0.525 (*p* < 0.01) for VOI_2_. At 4 cm spacing, *r*^2^ was 0.690 (*p* < 0.01) for VOI_1_ and 0.841 (*p* < 0.01) for VOI_2_. At 5 cm spacing, *r*^2^ was 0.762 (*p* < 0.01) for VOI_1_ and 0.859 (*p* < 0.01) for VOI_2_. Values obtained for ∆CBV through TRS and PET were positively correlated with each other; however, the strength of the relationship depended on both the optode distance and VOI used, and many of these values failed to reach statistical significance. At 2 cm spacing, *r*^2^ was 0.331 (*p* > 0.05) for VOI_1_ and 0.050 (*p* > 0.05) for VOI_2_. At 3 cm spacing, *r*^2^ was 0.633 (*p* < 0.05) for VOI_1_ and 0.277 (*p* < 0.01) for VOI_2_. At 4 cm spacing, *r*^2^ was 0.699 (*p* < 0.05) for VOI_1_ and 0.457 (*p* > 0.05) for VOI_2_. At 5 cm spacing, *r*^2^ was 0.585 (*p* > 0.05) for VOI_1_ and 0.352 (*p* > 0.05) for VOI_2_.	Small sample size No bias assessment was performed

* These studies had an initial sample size of 10 or greater; however, fewer than 10 individuals were included in the relevant analysis. AUTC, area under the curve; CA, contrast agent; CBF, cerebral blood flow; CBV, cerebral blood volume; CBVI, cerebral blood volume index; CBV_NIRS_, cerebral blood volume measured by near-infrared spectroscopy; CPAP, continuous positive airway pressure; CT, computed tomography; CW NIRS, continuous wave near-infrared spectroscopy; GL_P_, peak grey level; Hb, hemoglobin; ICG, indocyanine green; MRI, magnetic resonance imaging; NIRS, near-infrared spectroscopy; PET, positron emission tomography; RBC, red blood cells; RISA, radiodinated serum albumin; SaO_2_, oxygen saturation; SDS, source–detector distance; TD NIRS, time domain near-infrared spectroscopy; TRS, time-resolved spectroscopy; VOI, volume of interest.

## Appendix A

**Table A1:** PRISMA ScR checklist.

Section	Item	PRISMA-ScR Checklist Item
Title	1	See pg. 1
Abstract		
Structured summary	2	See pg. 1 (Abstract)
Introduction		
Rationale	3	See pg. 2 (Section 1)
Objectives	4	See pg. 3 (Section 1)
Methods		
Protocol and registration	5	N/A
Eligibility criteria	6	See pg. 3 (Section 2.2)
Information sources	7	See pg. 4 (Section 2.3)
Search	8	See pg. 4 (Section 2.3)
Selection of sources of evidence	9	See pg. 4 (Section 2.4)
Data charting process	10	All relevant data were collected from each article by primary author
Data items	11	See pg. 4 (Section 2.5)
Critical appraisal of individual sources of evidence	12	N/A
Summary measures	13	N/A
Synthesis of results	14	All data items for each method were tabulated and are included in Table 1
Risk of bias across studies	15	All articles published in academic journals, as such, biases were assumed to have been screened
Additional analyses	16	N/A
Results		
Selection of sources of evidence	17	See pg. 4–5 (Section 3)
Characteristics of sources of evidence	18	See Table 1
Critical appraisal within sources of evidence	19	N/A
Results of individual sources of evidence	20	See Table 1
Synthesis of results	21	All articles included outlined valid CBV measurement methods
Risk of bias across studies	22	All articles published in academic journals, as such, biases were assumed to have been screened
Additional analysis	23	N/A
Discussion		
Summary of evidence	24	See pg. 19–20 (Section 4)
Limitations	25	See pg. 20–22 (Section 4.1, Section 4.2 and Section 4.3)
Conclusions	26	See pg. 22 (Section 5)
Funding	27	See pg. 23 (Funding)

## Appendix B

The detailed strings of keywords that were used as search parameters in BIOSIS, SCOPUS, EMBASE, MEDLINE, Global Health, and Cochrane Library were as follows:

“brain” OR “cerebral” OR “cerebrum” OR “neuro” OR “encephalon”

ND

“NIRS” OR “near infrared spectroscopy” OR “near infrared spectroscopies” OR “NIR spectroscopy” OR “NIR spectroscopies” OR “NIR” OR “near infrared light” OR “rSO2” OR “regional cerebral oxygen saturation” OR “OxHgB” OR “oxygenated hemoglobin” OR “deOxHgB” OR “deoxygenated hemoglobin” OR “tHgB” OR “total concentration of hemoglobin” OR “CBF NIRS” OR “cerebral blood flow NIRS” OR “cerebral blood flow derived with near infrared spectroscopy” OR “TOI” OR “tissue oxygen index” OR “pCBF” OR “pulsatile cerebral blood flow” OR “HbO” OR “HHb” OR “THI” OR “total hemoglobin index” OR “total Hb index” OR “cerebral tissue oxygen saturation” OR “deoxyhemoglobin” OR “oxyhemoglobin” OR “regional oxygen saturation” OR “cerebral oxygen saturation” OR “cerebral oxygenation” OR “brain oxygen saturation” OR “near-infrared spectroscopy” OR “near-infrared spectroscopies” OR “near-infrared light”

AND

“CBV” OR “cerebral blood volume” OR “ICP” OR “intracranial pressure” OR “rCBV” OR “relative cerebral blood volume” OR “cerebral perfusion” OR “brain blood volume” OR “blood volume in brain tissue” OR “brain tissue blood volume” OR “pressure reactivity index” OR “PRx” OR “pulsatile CBV” OR “pulsatile cerebral blood volume” OR “AMP” or “fundamental amplitude of ICP” OR “fundamental amplitude of intracranial pressure” OR “C15O” OR “15 O-labeled carbon monoxide” OR “concentration-time curve”
